# Prostate transmembrane androgen inducible protein 1 *(PMEPA1)*: regulation and clinical implications

**DOI:** 10.3389/fonc.2023.1298660

**Published:** 2023-12-20

**Authors:** Qicui Zhu, Yue Wang, Yaqian Liu, Xiaoke Yang, Zongwen Shuai

**Affiliations:** ^1^ Department of Rheumatology and Immunology, The First Affiliated Hospital of Anhui Medical University, Hefei, China; ^2^ Inflammation and Immune Mediated Diseases Laboratory of Anhui, Hefei, China

**Keywords:** prostate transmembrane androgen-inducible protein 1 (*PMEPA1*), transcriptional regulation, Epithelial-mesenchymal transition (EMT), biomarker, lysosome

## Abstract

Prostate transmembrane androgen inducible protein 1 (PMEPA1) can promote or inhibit prostate cancer cell growth based on the cancer cell response to the androgen receptor (AR). Further, it can be upregulated by transforming growth factor (TGF), which downregulates transforming growth factor-β (TGF-β) signaling by interfering with R-Smad phosphorylation to facilitate TGF-β receptor degradation. Studies have indicated the increased expression of PMEPA1 in some solid tumors and its functioning as a regulator of multiple signaling pathways. This review highlights the multiple potential signaling pathways associated with PMEPA1 and the role of the *PMEPA1* gene in regulating prognosis, including transcriptional regulation and epithelial mesenchymal transition (EMT). Moreover, the relevant implications in and outside tumors, for example, as a biomarker and its potential functions in lysosomes have also been discussed.

## 1 Introduction

Prostate transmembrane androgen-inducible protein 1 (*PMEPA1*) is also called transmembrane prostate androgen–induced protein (*TMEPAI*) or solid tumor associated gene 1 (*STAG1*). It was first identified as an androgen-inducible gene in hormone-responsive androgen-responsive prostate cancer (LNCaP) cells. In that study, the analysis of a complementary deoxyribonucleic acid (cDNA) clone comprising an 1141 bp sequence from the *PMEPA1* gene revealed a 759 nucleotide open reading frame (ORF) encodeing a 252 amino acid protein with a predicted molecular mass of 27.8 kDa (1). It is located on chromosome 20q13.31-q13.33 and encodes five protein isoforms (-a -b -c -d -e), which are mainly expressed on cell membranes and subcellular organelles such as lysosomes and the Golgi apparatus (2–4). These are transmembrane proteins that contain SIM (a sequence that interacts with Smad) and their expression is mediated by androgens and transforming growth factor-β (TGF-β) (5). PMEPA1 contains the following three structural domains: N-terminal, transmembrane, and cytoplasmic structural domains. The transmembrane and cytoplasmic structural domains show highly conserved amino acid sequences, while the N-terminal structural domain exhibits length polymorphism in distinct heterodimers (3). Interestingly, only the PMEPA1-c isoform contains a truncated transmembrane structural domain of 13 amino acids and lacks the N-terminal structural domain. Further, while types a, b, d, and e have a type of perforation structure at the N-terminus that attaches to the Golgi apparatus, the c-type lacks this perforation structure (6). There is a PY motif (PPXY) in the cytoplasmic structural domain of *PMEPA1*, which binds to the four members of the E3 ubiquitin ligase homolog of the E6AP carboxy-terminal structural domain (2). PMEPA1 has been suggested to act as a regulator of multiple signaling pathways. For instance, it has been demonstrated to induce other signaling cascades such as mutated P53, Hippo, Wnt, and epidermal growth factor (EGF) pathways by interfering with tumorigenesis. These have been detailed in **Table 1** (1, 3, 7, 8, 12–14, 20). Additionally, it also plays roles in the cell cycle, cell proliferation, EMT, and other processes (14, 16, 17, 19, 21–23). In this review, we discuss the associated signaling pathways, and focus on the role exerted by the *PMEPA1* gene in prognosis. This includes transcriptional regulation, EMT regulation, and its clinical significance, both as a tumor biomarker and in applications beyond oncology.

**Table 1:** The implications of *PMEPA1* gene isoforms in different signaling pathways.

Signaling pathway	PMEPA1 isoform	Possible mechanisms	Reference
P53	Uncertain	To mediates p53-dependent apoptosis.	(7)
Wnt	Uncertain	As a possible downstream target for Wnt signaling, Wnt/β-catenin/TCF7L2 pathway can preferentially alter the transcriptional regulation of PMEPA1; To inhibit Wnt signaling by interfering with β-catenin stability and nuclear translocation; PMEPA1 plays an oncogenic role by activating the Wnt/β-catenin signaling pathway.	(8–10)
ROS/IRS-1	*PMEPA1*-d	Contributing to TGF-β1-induced EMT through ROS expression in lung cancer cells.	(11)
EGF	Uncertain	To be a possible direct molecular target in the EGF pathway in carcinogenesis process of breast and ovarian cancers. EGF signaling collaboratively regulates TGF-β-induced PMEPA1 expression.	(12, 13)
HIPPO	*PMEPA1*-a	Contributing to glioma progression through a dysregulation of the Hippo signaling pathway.	(14)
NF-κb	*PMEPA1*-a	NF-κb signaling participated in *PMEPA1*-induced cell proliferation and tumor growth in immunodeficient mice.	(15)
JNK	Uncertain	To enhance non-small cell lung cancer progression via activating the JNK signaling pathway.	(16)
MAPK	Uncertain	Stimulate proliferation and colony formation of pancreatic cancer cells through the MAPK signaling pathway; induced by RANK-p38 MAPK pathway signaling to upregulate cell surface expression of RANK, thus involving in osteoclastogenesis and osteoclast signaling.	(17, 18)
BMP	Uncertain	Activate BMP signaling, thereby promoting EMT and accelerating the proliferation and metastasis of colorectal cancer.	(19)

BMP, bone morphogenetic protein; EGF, epidermal growth factor; EMT, epithelial-mesenchymal transition; IRS-1, insulin receptor substrate-1; JNK, c-Jun N-terminal kinase; MAPK, mitogen-activated protein kinase; ROS, reactive oxygen species; TGF-β, transforming growth factor-β.

## 2 Regulation of PMEPA1-related signaling pathways

### 2.1 TGF-β signaling pathway

TGF-β is a well-known growth factor with an antiproliferative functioning. It plays a vital role in various physiological activities such as cell differentiation, apoptosis, cell migration, angiogenesis, matrix protein production, tumor promotion, and immunosuppression (24). It has been reported that PMEPA1 functions as a TGF-β regulator in prostate and non-prostate solid tumors. Further, the biological role of PMEPA1 subtypes in cancer has been comprehensively examined (3, 4, 11, 25–32). The inhibitory mechanisms of TGF-β signaling involved in PMEPA1 in prostate cancer are mainly applicable to subtype-a (3). Additionally, in androgen receptor (AR)-negative prostate cancer cells, the suppressive mechanisms are related to subtype d and e. The isoform d promotes the growth of cancer cells in a TGF-β-dependent manner while isoform e promotes in a TGF-β-independent manner, and both of them have no effects on AR-positive prostate cancer cells. Subtype-c does not influence prostate cancer growth and TGF-β signaling pathways, which suggests the importance of the N-terminal extracellular structural domains in different isoforms of *PMEPA1* (4). In breast and lung cancers, the suppressive mechanisms of TGF-β signaling are mainly suitable for subtypes a and d. These boost tumorigenic activity by inhibiting Smad phosphorylation levels and reducing the growth inhibitory effects of TGF-β/Smad signaling (25–28).

Studies have reported that *PMEPA1-a* inhibits the TGF-β signaling pathway through various steps (33). Additionally, TGF-β exerts regulatory control on PMEPA1 by modulating the ratio of Smad proteins and the converse is also true. To elaborate, PMEPA1 expression may be positively and negatively regulated by Smad3 and Smad2 separately, which suggests that the Smad3-PMEPA1 axis may be involved in reversing the growth-inhibitory effect of TGF-β to a growth-promoting function (34). To summarize, PMEPA1 isoforms are involved in the modulation of TGF-β, and the specific mechanisms need further study.

### 2.2 AR signaling pathway

The *PMEPA1*-*b* isoform has been identified as an androgen-responsive isoform and is abundant in the prostate (6, 31). Previous studies have shown that *PMEPA1*-*b* has different roles in the AR-positive and AR-negative prostate cancer tumorigenicity. In the AR-positive prostate cell line LNCaP, *PMEPA1*-*b* mediates AR protein degradation to inhibit prostate cancer proliferation in a proteasome-dependent manner by recruiting the E3 ubiquitin ligase NEDD4 (6, 31). Upon the inhibition of *PMEPA1-b*, the depletion of *PMEPA1-b* uncouples NEDD4 from AR degradation. The increased level of NEDD4 is available for phosphatase and tensin homolog (PTEN) degradation, leading to the simultaneous acquisition of AR and loss of PTEN in CaP cells. This further activates the PI3K/AKT signaling pathway and promotes tumor development (35). In another report, *PMEPA1-b* increased the proliferation of AR-negative RWPE1 and PC-3 prostate cells by inhibiting the Smad3/4-c-Myc-p21 signaling pathway (21). It was noted that *PMEPA1-e* promoted the growth of AR-negative prostate cancer cells in a TGF-β-independent manner while having no impact on AR-positive prostate cancer cells (4).

### 2.3 PI3K/AKT signaling pathway

**Figure 1:**
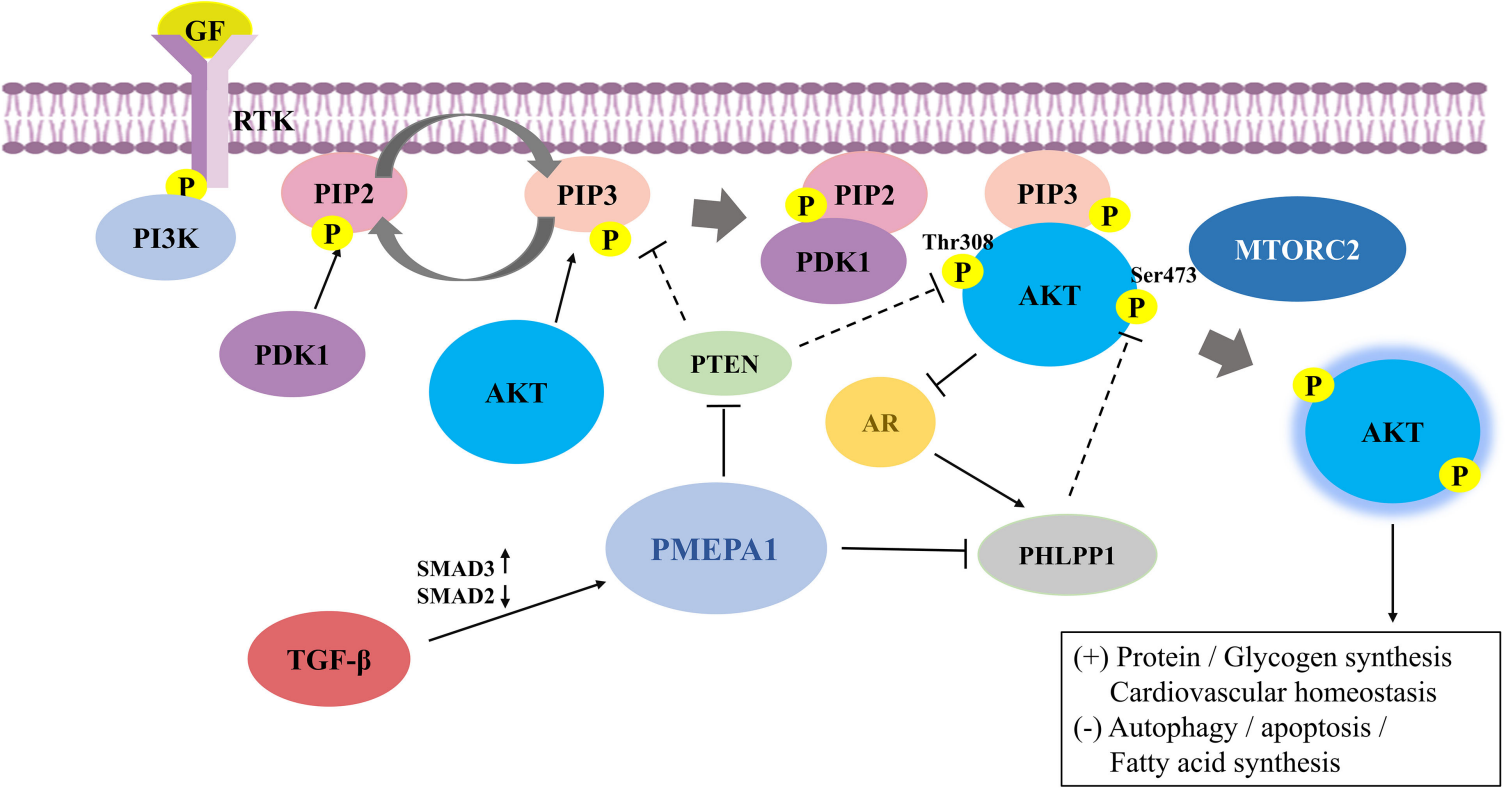
The mechanisms and the crosstalk of increased PI3K/AKT signaling by prostate transmembrane androgen-inducible protein 1 (PMEPA1). RTK binds to extracellular ligands that recruit PI3K to anchored receptors at the plasma membrane to activate PI3K signaling. PI3K phosphorylates PIP2 to PIP3, where the latter can be dephosphorylated to PIP2 in the presence of PTEN. PIP3 then binds to downstream effectors, including PDK1 and AKT, and recruits them to the membrane. PDK1 and mTORC2 phosphorylate AKT at the activation loop (Thr308) and hydrophobic group (Ser473), respectively, to completely activate AKT. PI3K/AKT signaling is also tightly regulated by negative regulatory molecules. PTEN suppresses PI3K/AKT signaling by inhibiting AKT activation and converting PIP3 to PIP2. Additionally, PHLPP1 dephosphorylates AKT at Ser473, leading to blocked AKT signaling. PMEPA1 promotes expression of the PI3K/AKT signaling pathway by increasing PTEN turnover and attenuating PTEN expression and mediates downregulation of PHLPP1 via the PY motif. AKT is cross-regulated with TGF-β and AR signaling in several mutually inhibitory loops. Further, TGF-β exerts a regulatory influence on PMEPA1 by modulating the ratio of Smad proteins. AKT phosphorylates AR at Ser-210 and inhibits the transcriptional activation of AR. Conversely, AR promotes PHLPP1 expression to stimulate AKT signaling. PI3K/AKT, phosphatidylinositol 3-kinase/protein kinase B; PMEPA1, prostate transmembrane androgen-inducible protein 1; RTK, Receptor tyrosine kinase; PIP3, phosphatidylinositol-3,4,5-trisphosphate; PIP2, phosphatidylinositol-4,5-bisphosphate; PDK, phosphatidylinositol-dependent kinase; mTORC2, mammalian rapamycin complex 2; PTEN, phosphatase and tensin homolog; PHLPP1, pleckstrin homology domain leucine-rich repeat protein phosphatase 1; TGF-β, transforming growth factor-β; AR, androgen receptor.

A study examining the role of adriamycin in triple-negative breast cancer cells (TNBC) suggested that PMEPA1-positive cells had a higher phosphorylation profile of PI3K and AKT (22). Several different molecular mechanisms have been established of how PMEPA1 regulates the PI3K-AKT-mTOR signaling pathway (summarized in **Figure 1**). As mentioned earlier, PTEN is a lipid phosphatase that is considered to be the major dose-dependent tumor inhibitor in the PTEN/PI3K/AKT signaling circuit (36). For instance, knockdown of *PMEPA1* could enhance the basal levels of PTEN in MDA-MB-231, although the mechanism remained unclear (25). It has been reported that PMEPA1 could exacerbate triple-negative breast cancer (TNBC) tumor progression by increasing PTEN turnover and attenuating PTEN expression to promote the expression of atypical PI3K/AKT signaling. Further, down-regulation of *PMEPA1* could increase PTEN expression and reduce PI3K/AKT expression, and reduced breast cancer tumor volume (37). Furthermore, Yang Y et al. found that *PMEPA1* interference could effectively inhibit the proliferation, aggression, and migration of pancreatic cancer cells. Furthermore, this interference could promote PTEN expression, thus enhancing the sensitivity of human pancreatic cancer (hPAC) cells to gemcitabine (GEM) and cisplatin (DDP) by inhibiting the PI3K/AKT signaling (38). Additionally, an *in vitro* study suggested that *PMEPA1* mediated the downregulation of pleckstrin homology domain leucine-rich repeat protein phosphatase 1 (PHLPP1) through the PY motif. This promoted the phosphorylation of Ser473 of AKT and mediated PI3K/AKT signaling, subsequently leading to tumor progression in TNBC. Further, NEDD4-2 was found to be an essential E3 ligase regulating PHLPP1, which was involved in mediating PMEPA1-induced degradation of the PHLPP1 proteasome (39). These findings suggest that PMEPA1 may enhance tumorigenicity through the PI3K signaling pathway.

### 2.4 Crosstalk among signaling pathways and PMEPA1

The TGF-β pathway has been reported to crosstalk with multiple signaling cascades related to PMEPA1. A study revealed that ELK-1, a downstream molecule of the EGF pathway, bound to the first intron of the PMEPA1 gene together with Smad3 activated by TGF-β, and enhanced PMEPA1 transcription (13). A decrease in Smad3 levels not only reduced TGF-β-induced levels of PMEPA1 but also increased PTEN expression, which reduced AKT activation in the presence of TGF-β (34).

## 3 The role of *PMEPA1* gene in disease prognosis

### 3.1 Transcriptional regulation of *PMEPA1* gene

Studies over the past two decades have provided crucial information on the transcriptional regulation of *PMEPA1* in disease development. For instance, a study established that methylation of the first intron deoxyribonucleic acid (DNA) region of the *PMEPA1* gene is a prevalent mechanism of *PMEPA1* silencing in human prostate CaP, which was essential in regulating AR stability (40). Another study found that hypermethylation of CpG at different sites was related to regulation in gene expression and disease prognosis (41, 42). Similar results were observed in research published by Kulis et al, where alterations in DNA methylation in both intergenic regions and intragenic regions are regulated in development and disease. Further, these alterations were found to be actively involved in the regulation of transcription (43). In recent years, an increasing number of researchers have focused on the prognostic values of DNA methylated sites. For instance, Li and Kong reported that methylation of eight CpG sites was negatively regulated by *PMEPA1* DNA methylation in cervical cancer (CC) specimens. Among these, cg17482197 and cg08583507 with elevated standard were related to poor prognoses of CC patients. This suggests that hypermethylation of *PMEPA1* CpG sites can result in the downregulated expression of *PMEPA1* and may be an independent prognosis indicator for CC patients (23). In addition, another study on the *PMEPA1* promoter demonstrated that methylation of the specificity protein 1 (SP1) binding site may contribute to *PMEPA1* gene repression (44). Promoters were reported to be co-regulated by TGF-β/Smad and Wnt/β-linked protein/T cell factor 7L2 (TCF7L2) (8, 45). Furthermore, it is identified that EGF signaling significantly enhanced TGF-β -induced *PMEPA1* expression. The mechanism entails the binding of ELK-1 to the first intron (+1037 to +1294) of the *PMEPA1* together with TGF-β activated Smad3, which leads to the coordinated activation of *PMEPA1* gene transcription (13). Another finding that Smad3 positively regulated TGF-β-induced *PMEPA1* promoter activity while Smad2 had a negative control on this activity also strengthened this argument (34).

To better understand the functions of *PMEPA1*, the post-transcriptional regulatory mechanisms have also been investigated. A published report showed that miR-19a-3p potentially targets the 3’-untranslated region (UTR) *PMEPA1* at nucleotides 359-366 and 611-617, and siRNA-mediated knockdown of *PMEPA1* leads to a higher proliferation, migration, and invasion of PCa cells, indirectly demonstrating that elevated miR-19a-3p expression leads to tumor progression (46). A novel linc00941 was identified in esophageal squamous cell carcinoma (ESCC), which was suggested to be a competing endogenous RNA by bioinformatics prediction and tests for miR-877-3p, which targeted the 3’ UTR of *PMEPA1*, thereby inhibiting *PMEPA1* expression (47). Further, miR-129-5p was also identified as a miRNA that repressed *PMEPA1* expression in melanoma. Additionally, CSDE1, an RNA-binding protein (RBP), which promotes metastasis along with miR-129-5p/AGO2 miRNA-induced silencing complexes (miRISC), negatively regulated *PMEPA1* expression (48). A dual-luciferase reporter assay verified that miR-130a-3p has a binding site for the 3’UTR of *PMEPA1*, and inhibits the secretion of estradiol and progesterone in granulosa cells (GCs) by targeting *PMEPA1* (49). These findings summarize a few transcriptional and post-transcriptional regulatory mechanisms related to the *PMEPA1* gene, including the effects of DNA methylation, promoters, and non-coding RNAs on *PMEPA1* transcription. These observations deepen our understanding of the molecular mechanisms of genes and provide a theoretical basis for disease prognosis.

### 3.2 Regulation of PMEPA1 in EMT

EMT is a fundamental biological event that is essential in embryonic development, chronic fibrosis, and cancer progression (50, 51). EMT can induce a transition of epithelial cells to a mesenchymal state and fundamental changes in cell behaviors and properties, including migration, invasion, and proliferation, which are key steps in cancer progression (52, 53). During EMT, the expression of epithelial markers (E-calmodulin) decreases while the levels of mesenchymal markers (vimentin and fibronectin) increase. These markers are regulated by Snail, ZEB, and Twist by blocking the CDH1 gene encoding E-calmodulin (54).

Extensive studies have confirmed that PMEPA1 is strongly correlated with the expression of markers and transcription factors associated with EMT, while EMT also influences the proliferation and migration of tumor cells. For instance, Hu et al. examined five human lung cancer cell lines and found that *PMEPA1* is strongly expressed in cells with relatively elevated levels of mesenchymal characterization. Further, immunofluorescence staining of F-actin showed that *PMEPA1* silencing could lead to a decrease in F-actin reorganization during EMT in lung cancer cells (11). In another study, Prajjal et al. showed that Smad3 deficiency was related to a reduction in the expression of EMT-inducing transcription factors and E-cadherin, while the expression of cell cycle inhibitors and vimentin was increased. The decreased growth, invasion, and associated gene expressions were primarily reversed by overexpressing *PMEPA1* in Smad3 knockdown cells, which suggests that *PMEPA1* is a key factor involved in EMT (34). A loss-of-function study demonstrated that doxorubicin significantly increases levels of EMT transcription factors studied in *PMEPA1*-positive cells but not in *PMEPA1* knock-out cells (22). Further, Lei et al. demonstrated that down-regulation of *PMEAP1* increased the expression of E-cadherin and *PMEPA1* enhanced EMT-mediated metastasis by targeting the bone morphogenetic proteins (BMP) signaling cascade (19).. A study conducted by Alba et al. showed different results where overexpression of *PMEPA1* resulted in an elevated expression of E-cadherin and also induced Vimentin and ZEB1 expression. The cause of the differences in E-cadherin expression was not attributed to the absence of CDH1 inhibition, but might be dependent on TGF-β signaling (55). Zou et al. demonstrated that silencing of *PMEPA1* significantly inhibited the migratory ability of MDA-MB-231 cells and facilitated the mesenchymal-epithelial transition process in breast cancer cells. Further, overexpression of *PMEPA1* promoted cell migration and maintained the mesenchymal-like morphology of cancer cells (56). Another study conducted in triple-negative breast cancer tumor samples reported that *PMEPA1* knockdown reduced Snail levels and inhibited cell migration, invasion, and metastasis both *in vitro* and *in vivo* (37). Studies based on bioinformatics analysis and *in vitro* assays showed that *PMEPA1* played a role in bladder cancer (BLCA) progression and the tumor microenvironment (TME) (57). Moreover, the miR-877-3p/*PMEPA1* axis plays a vital role in accelerating esophageal squamous cell carcinoma (ESCC) cell proliferation, metastasis, and EMT in esophageal cancer (47). Similar results were also observed in idiopathic subglottic stenosis (iSGS) patients, where *PMEPA1* was identified as an EMT regulator and, its high expression is related to a shorter recurrence interval of the disease (58).

**Figure 2:**
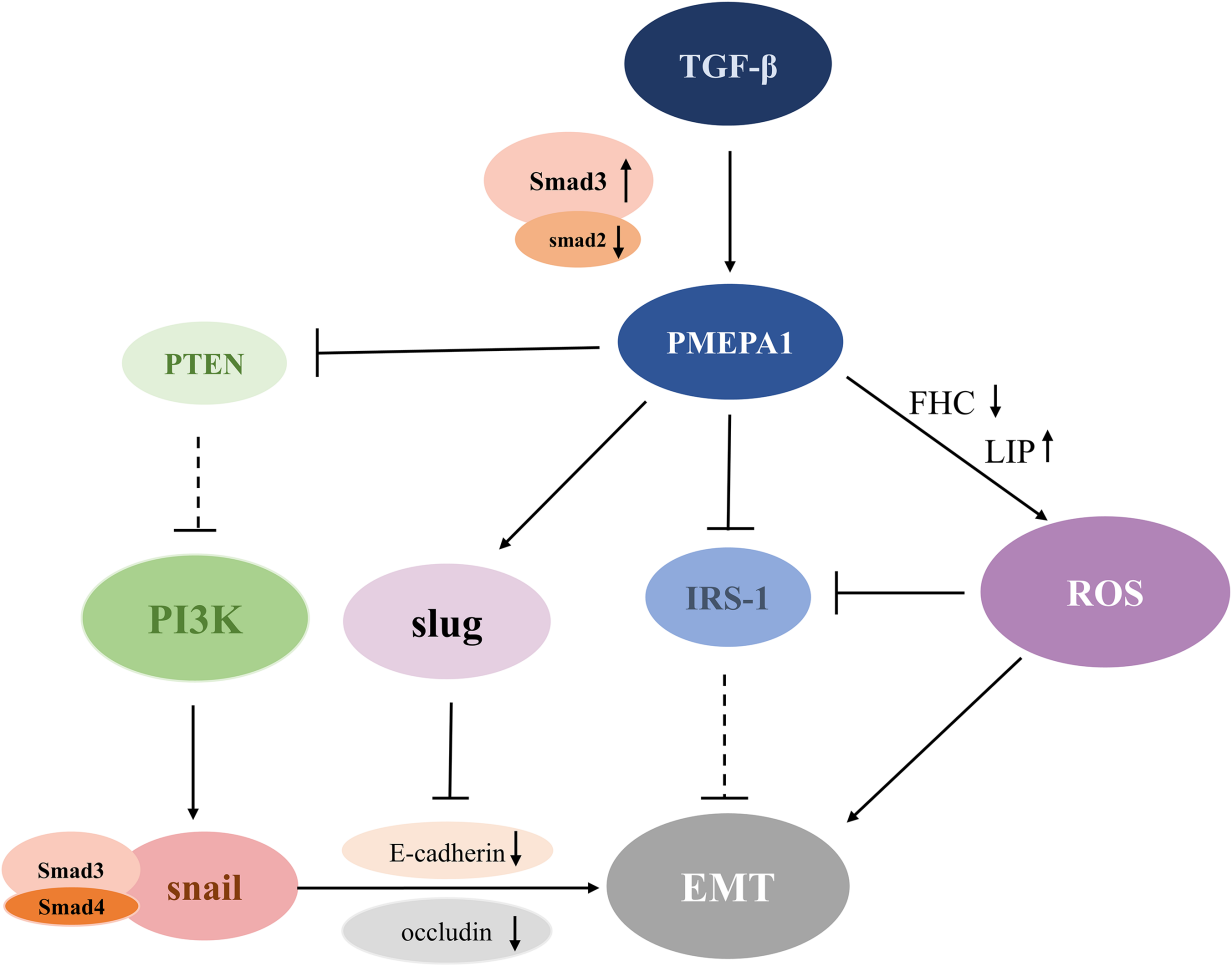
Schematic diagram of the PMEPA1-mediated EMT pathway. TGF-β stimulates the expression of PMEPA1, which directly down-regulates the levels of IRS-1 and FHC. This further promotes LIP conduction and leads to increased ROS production and EMT. Additionally, PMEPA1 promotes the production of Slug, which binds to the E-box region to inhibit the expression of E-cadherin and augment EMT formation. Increased expression of Smad3 or reduced expression of Smad2 upregulates the level of PMEPA1, which increases PTEN degradation of PTEN, and diminishes its suppressive effect on the PI3K signaling pathway. The activated PI3K signaling pathway induces EMT by downregulation of E-cadherin and occludin through a Snai1/Smad3/Smad4 complex. TGF-β, transforming growth factor-β; PMEPA1, prostate transmembrane androgen-inducible protein 1; IRS-1, insulin receptor substrate-1; ROS, reactive oxygen species; PTEN, phosphatase and tensin homolog; PI3K, phosphatidylinositol 3-kinase; EMT, epithelial-mesenchymal transition; FHC, ferritin heavy chain; LIP, labile iron pool.

Many molecular mechanisms have been established to support the modulation of EMT by *PMEPA1*. The detailed regulatory mechanism is shown in **Figure 2**. A study conducted in human lung cancer cells revealed that *PMEPA1* contributes to TGF-β-induced EMT by the downregulation of insulin receptor substrate-1 (IRS-1) and the production of reactive oxygen species (ROS) (11). Recent studies reported that ROS factors can trigger the development of EMT (59). Further, *PMEPA1* enhances TGF-β-induced downregulation of ferritin heavy chain (FHC), increasing the expression of the labile iron pool (LIP), thus boosting the production of ROS and EMT (59). Additionally, IRS-1 is known to be an EMT suppressor that plays a crucial role in maintaining the epithelial phenotype. Interestingly, TGF-β induced a significant increase in intracellular ROS levels in A549 cells, which decreased the expression of the EMT inhibitory factor IRS-1, thereby contributing to EMT progression (60). It was also demonstrated that *PMEPA1* knockdown suppressed the induction of the E-cadherin transcriptional repressor Slug, which repressed E-cadherin expression by binding to the E-box region of the E-cadherin promoter (61). Additionally, the TGF-β-SMAD3-PMEPA1-PTEN axis may be involved in regulating EMT. For instance, PMEPA1 negatively regulated PTEN protein levels in TNBC cell lines to activate the PI3K signaling pathway. This subsequently activated EMT-related Snai1 by influencing Snai1/Smad3/Smad4 complex-mediated low expression of E-cadherin and occludins (62, 63). To summarize, PMEPA1 plays a central role in EMT-related signaling pathways and the TGF-β signaling pathway that promotes late tumor metastasis.

## 4 Implications of *PMEPA1*

### 4.1 As a potential prognostic biomarker

A previous study revealed that PMEPA1 isoforms (a, b, and d) may be a surrogate for androgen and TGF-β signaling in prostate cancer to serve as a biomarker for monitoring disease progression and aggressive clinical outcomes (3, 4). Further, PEMPA1 was identified as a hepatocellular carcinoma (HCC) classifying factor with a late TGF-β signature that accurately predicted liver metastasis in a genome-wide mouse HCC microarray study (64). Bioinformatics analysis revealed that PMEPA1 may be a new potential biomarker for predicting disease progression and prognosis of BLCA (57). Further, univariate and multivariate analyses showed that PMEPA1 expression was an independent predictor of overall survival in patients with CC, and its increased expression was positively correlated with elevated levels of immune infiltration of a variety of immune cells (23). Survival analyses of the adenocarcinoma of lung cancer (LUAD) and squamous cell carcinoma of lung cancer (LUSC) cohorts indicated that patients with a greater expression of PMEPA1 had shorter disease-free survival compared to patients with a lower expression of PMEPA1 (p<​0.05) (16). These results supported the observations of survival analyses outlining how PMEPA1 was related to the prognosis of both LUAD and LUSC (65, 66). Shen et al. developed a predictive model of eight genes related to gastric adenocarcinoma (GA) chemotherapy, including PMEPA1, through univariate Cox and LASSO regression analyses. The model showed great validity in predicting the prognosis of GA patients within 1-5 years (67). Taken together, survival analysis further sheds light on the potential of PMEPA1 to be a novel biomarker in predicting tumor progression and prognosis.

### 4.2 The role of PMEPA1 in lysosomes

#### 4.2.1 Localization and transport mechanisms of PMEPA1

PMEPA1 is localized to the lysosome and Golgi apparatus and is translocated to the lysosome depending on binding to the E3 ubiquitin ligase NEDD4, which is the molecular basis for linkage to the lysosome. The molecular mechanisms of the transport of PMEPA1 to the lysosome are unclear (68, 69). It was observed that clathrin and the cationic non-dependent mannose-6-phosphate receptor (CI-M6PR) mediated the direct transport of PMEPA1 from the Golgi to endo-lysosomes. Further, the ubiquitination of PMEPA1 modified the signal for lysosomal transport, and the ubiquitin-binding proteins Hrs and STAM were involved in its lysosomal transport. In addition, the aa132–155 domain of PMEPA1 was involved in its interaction with Dynactin 5 and Dynactin 6 in lysosomal trafficking (69). Additionally, it was reported that lysosomal-associated protein transmembrane 5 (LAPMT5), a lysosomal membrane protein, was sorted from the Golgi to the lysosome by association via its PY motifs with Nedd4. It was hypothesized that it was associated with PMEPA1 transportation (70).

#### 4.2.2 Regulation of PMEPA1 in osteoclasts

Osteoclasts are multinucleated cells formed by the fusion of osteoclasts, which originate from the hematopoietic monocyte/macrophage lineage. The osteoclast differentiation is promoted by macrophage colony-stimulating factor (M-CSF) and receptor activator of nuclear factor-κb (NF-κb) ligand (RANKL). Mature osteoclasts are activated on the bone surface to absorb bone (71).

**Figure 3:**
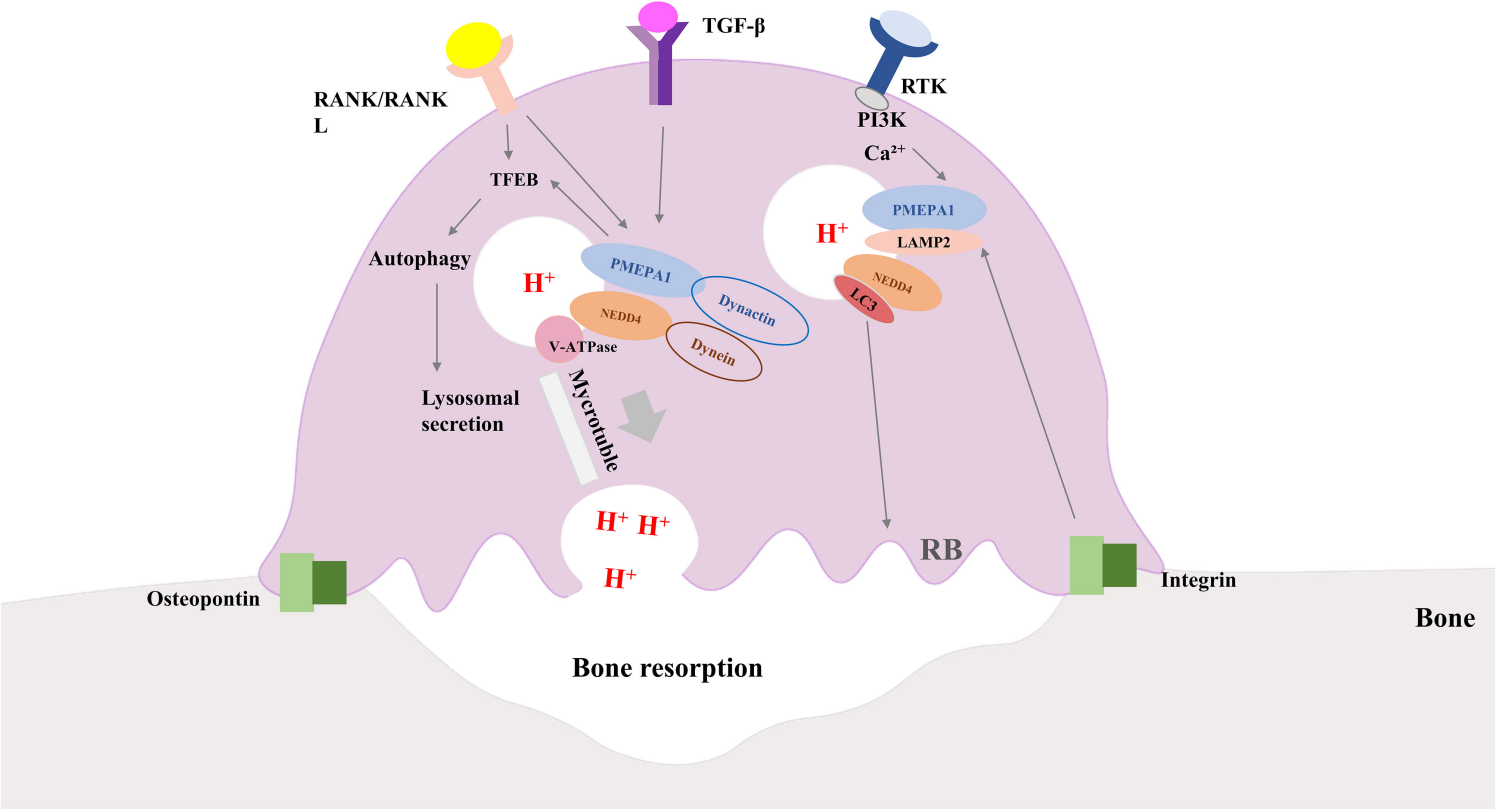
Diagram of how osteoclasts work along with the role of PMEPA1. Interaction of integrins and bone matrix leads to attachment of osteoclasts to bone, further inducing polarization of osteoclasts. RANK/RANKL signaling and PMEPA1 activate TFEB, which promotes autophagy and increases lysosomal secretion. PMEPA1 levels are increased by the PI3K signaling pathway and intracellular Ca2+, and the release of TGF-β and bone-bridging proteins from osteoclasts further increases the expression of PMEPA. PMEPA1 locates at LAMP-2, partially co-localizes with LC3 lysosomes, NEDD4 and V-ATPase components (V0A3, V0D2 and V1B2 subunits). PMEPA1 and NEDD4 interact with dynactin and dynein, respectively, and facilitate lysosomal movement along microtubules toward the plasma membrane to secrete protons. PMEPA1 also promotes bone resorption by regulating the formation of actin rings through LC3. RANK/RANKL, receptor activator of nuclear factor-kB/RANK Ligand; TGF-β, transforming growth factor-β; PI3K, phosphatidylinositol 3-kinase; PMEPA1, prostate transmembrane androgen-inducible protein 1; LC3, light chain 3; NEDD4, the neuronally expressed developmentally downregulated 4; TFEB, transcription factor EB; LAMP -2, lysosomal-associated membrane protein 2.

The mechanisms associated with *PMEPA1* in osteoclasts for bone resorption may include osteoclast production, differentiation, fusion, actin ring formation, vesicle transport, and bone signaling. An *in vitro* osteolysis assay demonstrated that histone deacetylase 3 (HDAC3) deficiency inhibited osteolysis, the number of nuclei per cell, and bone resorption. However, the knockdown of *PMEPA1* partially restored the osteogenesis defect caused by the HDAC3 deficiency, suggesting that osteoclast fusion might be regulated by the effect of HDAC3 on *PMEPA1* expression (72). Another study conducted in bone marrow macrophages and the osteoclast precursor cell line osteoclast-like cell (RAW-D) indicated that RANKL activated the PMEPA1 via the P38 pathway to promote osteoclast formation (18). Xu et al. performed *in vitro* experiments to further investigate the possible mechanisms of *PMEPA1* in bone resorption activity. They showed that the knockdown of *PMEPA1* expression impaired bone resorption activity and inhibited the formation of the ring-like, actin-abundant podosome belt which was essential for osteoclast function. Moreover, some of the PMEPA1 molecules co-localized with light chain 3 (LC3) where inhibition of PMEPA1 could reduce the size of the actin rings (73). It has been described earlier that LC3 could regulate actin ring formation and bone-resorbing capacity in osteoclasts (74). This led to the hypothesis that PMEPA1 might participate in the recombination of the actin skeleton and regulate the formation of the actin ring through LC3. ​Additionally, it has been proposed that LC3 regulates the lysosomal secretion of osteoclasts, which suggests that PMEPA1 is directly involved in lysosomal secretory functions in activated osteoclasts (75). More research is needed to corroborate these hypotheses. Osteoclasts produce protons and lysosomal enzymes such as cathepsin K via lysosomal secretion to digest the bone matrix through the ruffled border (RB), which highlights the vital role of intracellular vesicular trafficking in osteoclast functions (76, 77). For instance, Hirohito et al. investigated the mechanisms of PMEPA1 in intracellular vesicular trafficking and showed that PMEPA1 controlled proton secretion in osteoclasts by regulating vesicular transport through NEDD4. In this study, mutant mice lacking the NEDD4-binding structural domain of PMEPA1 exhibited enhanced bone volume and reduced bone resorption activity. Immunofluorescence analysis indicated that PMEPA1 co-localized with the NEDD4, V0A3, and V0D2 subunits of vesicular ATPase to regulate osteoclast proton production and that osteoclast proton secretion was significantly reduced in PMEPA1 mutant osteoclasts. To summarize, PMEPA1 and NEDD4 were found to be critical regulators of proton production. The specific regulatory mechanism has been illustrated in **Figure 3**.

#### 4.2.3 Regulation of PMEPA1 in autophagy

Autophagy is a cellular degradation pathway, in which autophagosomes merge with lysosomes to form autolysosomes to degrade all the lysosomal contents. Autophagy is triggered in the condition of starvation, hypoxia, and other unfavorable environments, while under normal conditions, it is present at low levels (78). It has been suggested that LC3 is involved in the regulation of microtubule assembly and disassembly (79). During autophagosome membrane formation, cytosolic LC3 (LC3-I) binds to phosphatidylethanolamine (PE) to form LC3-II via two ubiquitination reactions catalyzed by Atg7 and Atg3 (80–82). Lysosomal proteases degrade LC3-II within the autophagosome during the fusion of the autophagosome with lysosomes. Beclin-1 is the major mammalian autophagy-related gene, which can induce autophagy and inhibit tumorigenesis (83, 84). Recently, *PMEPA1* deficiency has been proven to cause lysosomal instability and inhibit autophagy. Furthermore, the inhibition of autophagy due to *PMEPA1* deficiency was associated with lysosomal membrane permeabilization and regulation of Beclin-1 transcriptional levels (85). A previous study also indicated that *PMEPA1* mutation and NEDD4 knockdown altered the expression of autophagy-related proteins (86). Furthermore, NEDD4 interacted with LC3 through an LC3-interacting motif to regulate autophagy in cancer cells. Hence, it was hypothesized that PMEPA1 might interact with LC3 through a NEDD4-mediated process to regulate lysosomal and influence autophagy (87). It was also noted that transcription factor EB (TFEB), which regulates autophagosome biogenesis, was down-regulated in PMEPA1 mutant osteoclasts. Hence, it was suggested that the effect of *PMEPA1* on autophagy is mediated by TFEB, which is regulated by RANK/RANKL signaling (86).

## 5 Discussion

The essential roles of PMEPA1 in associated signaling cascades and its prognostic value have been better understood by significant lab and clinical in-depth studies. As mentioned earlier, the inhibitory mechanisms of TGF-β signaling involved in PMEPA1 are mainly applicable to subtype-a. The suppressive mechanisms associated with subtype-d are related to the suppression of Smad promoter activity and are still under study (4). In addition to isoform-b, isoform-e also affects the AR signaling pathway. Though the mechanism does not modulate classical AR and TGF-β signaling in prostate tumorigenesis, it may be linked to a partial intron retention between exons two and four, maintaining the open reading frame. An additional understanding of the cell growth-promoting mechanisms of *PMEPA1*-e is needed (4). Further, PMEPA1 promotes the PI3K/AKT signaling pathway by increasing PTEN turnover and attenuating PTEN expression while mediating the downregulation of PHLPP1 via the PY motif. However, the specific mechanisms associated with the regulation of these PMEPA1 isoforms regulation in various signaling pathways are unclear. Further study of the “division of labor” of PMEPA1 isoforms can expand our understanding of the multiple functions of PMEPA1 in oncogenesis.

The crosstalk of PMEPA1 and signaling pathways is not well studied. The AKT and AR signaling have been revealed to cross-regulate in several reciprocal inhibitory loops. For instance, AKT phosphorylated AR at Ser-210, and subsequently inhibited AR transactivation (88). Further, AR inhibition can stimulate AKT signaling by decreasing the expression of the AKT phosphatase PHLPP1 (89, 90). Given that PMEPA1 may modulate the expression of PHLPP1 via the PY motif, it is hypothesized that inhibition of PMEPA1 may be involved in the crosstalk mechanism and can be beneficial for cancer therapy. However, further analyses of these specific crosstalk mechanisms need to be confirmed.

In addition to this, many signaling pathways are involved in the process of EMT. Accumulating evidence has suggested that the AKT pathway is closely related to the EMT process (91, 92). AKT activation directly causes a reduction in E-calmodulin and triggers Twist expression, which further reduces the leverage of E-calmodulin and inhibits cell migration. Activation of NF-κb via AKT may result in the accumulation of ZEB-1, represses E-calmodulin expression to accelerate EMT (93). Further, mTORC2 regulates the actin cytoskeleton of cells and EMT by modulating the phosphorylation state of protein kinase C (PKC) and Akt activation (94). However, there has been considerably less research on the direct crosstalk between EMT and PMEPA1 in AKT signaling. The close relationship between PMEPA1 and PI3K signaling pathways has been demonstrated previously, allowing for the hypothesis that PMEPA1 plays an integral role in the mechanisms underlying EMT through the PI3K signaling pathway. Additionally, TGF-β has been shown to trigger EMT by interacting with the regulatory p85 subunit of PI3K or directly activating the mTOR pathway (95–97). Regulation of Smad phosphorylation in the TGF-β pathway activates EMT transcription factors, including Snail, Twist, zeb1, and Slug, subsequently inducing Vimentin and suppressing E-cadherin (98).

As previously described, LC3 can regulate actin ring formation and bone-resorbing capacity in osteoclasts. Further, given that PMEPA1 co-localized with LC3, it is hypothesized that PMEPA1 might participate in the recombination of the actin skeleton and regulate the formation of the actin ring through LC3 (74). ​Additionally, it has been proposed that LC3 regulates the lysosomal secretion of osteoclasts, thus suggesting that PMEPA1 is directly involved in lysosomal secretory function in activated osteoclasts (75). Further studies are needed to confirm the hypotheses.

Although PMEPA1 is localized in the lysosome and has a strong correlation with autophagy-associated proteins, there are limited studies on PMEPA1 and autophagy. TGF-β is one of the most abundant growth factors in the bone and can be released in osteoclasts during bone resorption (99). An emerging body of evidence from studies in cells and experimental animal models has suggested that TGF-β can regulate autophagy (100–102). Hence, it will be of great value to clarify whether PMEPA1 controls the formation of autophagosomes through TGF-β signaling. Moreover, it has been established that autophagy has a dual role in tumor growth, survival, and metastasis, depending on the tumor stage, biology, and the surrounding microenvironment (103). It remains to be further investigated whether the tumor-promoting effect of PMEPA1 is regulated by autophagy. Therefore, whether autophagy is involved in the lysosomal secretion regulated by PMEPA1 needs to be probed further along with the detailed mechanism.

## 6 Conclusion

Accumulating pieces of evidence have demonstrated that the *PMEPA1* gene is a crucial regulator of tumor progression and multiple biological functions. This review summarizes and highlights specific functions of PMEPA1 isoforms in the context of TGF-β and AR signaling. The biological functions of PMEPA1 in the context of the PI3K/AKT pathway and the crosstalk between the various signaling pathways in cancer have also been covered. Moreover, the transcriptional regulation mechanisms of PMEPA1, its role in EMT modulation, and clinical applications in and outside cancer have also been discussed. While PMEPA1 isoforms a and d are the main isoforms that inhibit TGF-β signaling, the main isoform that inhibits AR signaling in prostate cancer cells is PMEPA1-b. PMEPA1 accelerates the progression of the PI3K signaling pathway by suppressing the expression of PHLPP1 and PTEN. Hypermethylation of CpG at different sites in PMEPA1 is related to disease prognosis, and gene-associated transcription is tightly regulated by microRNA and lincRNA. Further, the transcriptional regulation of PMEPA1 and its influence on EMT have an important impact on cancer prognosis. Given that PMEPA1 also contributes to bone resorption and lysosome-induced autophagy by interfering with a wide range of biological processes, it may serve as a reliable marker in both tumors and non-cancerous diseases. Further research into the pathogenesis of PMEPA1 can lead to novel ideas and discoveries, which will contribute to a better prognosis for patients with related diseases.

## Author contributions

QZ: Writing – original draft. YW: Writing – review & editing. YL: Writing – review & editing. XY: Supervision, Writing – review & editing. ZS: Conceptualization, Supervision, Writing – review & editing.
